# Improving Dialysis Adherence for High Risk Patients Using Automated Messaging: Proof of Concept

**DOI:** 10.1038/s41598-017-03184-z

**Published:** 2017-06-23

**Authors:** A. Som, J. Groenendyk, T. An, K. Patel, R. Peters, G. Polites, W. R. Ross

**Affiliations:** 1 Epharmix Research Center, Washington University School of Medicine, St. Louis, USA; 2 Saint Louis University School of Medicine, St. Louis, USA; 3 Division of Emergency Medicine, Washington University School of Medicine, St. Louis, USA; 4 Renal Division, Washington University School of Medicine, St. Louis, USA

## Abstract

Comorbidities and socioeconomic barriers often limit patient adherence and self-management with hemodialysis. Missed sessions, often associated with communication barriers, can result in emergency dialysis and avoidable hospitalizations. This proof of concept study explored using a novel digital-messaging platform, EpxDialysis, to improve patient-to-dialysis center communication via widely available text messaging and telephone technology. A randomized controlled trial was conducted through Washington University-affiliated hemodialysis centers involving ESRD patients with poor attendance, defined as missing 2–6 sessions over the preceding 12 weeks. A cross-over study design evaluated appointment adherence between intervention and control groups. Comparing nonadherence rates eight weeks prior to enrollment, median appointment adherence after using the system increased by 75%, and median number of unintended hospitalization days fell by 31%. A conservative cost-benefit analysis of EpxDialysis demonstrates a 1:36 savings ratio from appointment adherence. EpxDialysis is a low-risk, cost-effective, intervention for increasing hemodialysis adherence in high-risk patients, especially at centers caring for vulnerable and low-income patients.

## Introduction

End-stage renal disease (ESRD) is a major health issue, with more than 660,000 patients being treated for ESRD in the United States, according to the latest U.S. Renal Data Systems Annual Data Report^1^. Among those patients, 468,000 receive chronic hemodialysis (HD) therapy. Despite being a life-saving medical treatment, dialysis regimens which last on average 3–4 hours, three times per week, pose a significant burden on work and vocational activities^2, 3^. The demands of dialysis treatment, coupled with socioeconomic barriers encountered by the ESRD population, lead to significant treatment non-adherence^4–6^. Medical factors such as untreated depression, psychiatric illness, pain, and gastrointestinal discomfort also contribute to treatment noncompliance^7, 8^. Younger age and smoking are associated with increased nonadherence in hemodialysis^9^. Other factors influencing compliance include clinic size, clinic location, and demographic profile of the clinic patients^10^.

Missed or shortened hemodialysis treatment sessions are common, occurring between 7% and 32% among chronic HD patients^10, 11^. In a study involving 54 dialysis units, 5–7% of patients missed at least one treatment session within a 4-week window^12^. Over 12-week follow-up, one half of patients had missed or shortened treatments. Hemodialysis nonadherence has consequences such as bone demineralization, pulmonary congestion and electrolyte disturbances^13^. Missing >1 HD appointment per month is associated with a 30% increase in mortality, though the exact mechanisms of this increase are unclear^14^. Most likely, patients develop critical complications of ESRD with high risks to patient morbidity and mortality and require emergent dialysis and/or acute hospitalization at significant healthcare cost^15, 16^.

Missed appointments increase the risk of healthcare interventions. In the two days after a missed dialysis appointment, 5% of patients are hospitalized vs. 1.2% of those that attended the appointment. Similar trends have been demonstrated for emergency department (ED) presentation, intensive care unit (ICU) admission, and urgent in-hospital dialysis^16^.

Although not specific to a HD population, previous studies have shown that interventions such as electronic messages, telephone reminders, and family based contracts have improved attendance rates in a primary care setting^4^. In a study observing primary care outpatient appointment attendance, proactive computer-generated letter and phone reminders reduced the patient no-show rate by 10%^11^. Despite the availability of commercial smartphone applications for clinical appointment planning, the prevalence of smartphone devices among older, lower income patients, consistent with the ESRD population, is low^17^. Given these needs and constraints, automated electronic communication using ubiquitous forms of technology (e.g. landline phone, cell phone and text message) may be a cost effective and easily implemented solution for hemodialysis non-adherence.

The proof-of-concept study was conducted in the Chromalloy American Kidney Center (CAKC), located within Washington University School of Medicine at Barnes-Jewish Hospital, and its affiliated Forest Park Dialysis Unit, in close proximity to CAKC. CAKC treats approximately 200 patients, while the Forest Park unit serves 138 patients. Both dialysis centers are staffed by faculty from the Washington University Renal Division. The dialysis units serve a predominantly low-income, minority population from economically disadvantaged neighborhoods contiguous to the medical school. Approximately 65% of the patients are African American, and 25% of the clients receive Medicaid at the inception of their dialysis treatments. The demographic makeup and adverse payer mix of the dialysis units are distinct from the surrounding affluent, mostly White townships, so the results of this study may not be generalizable to suburban dialysis units or units not affiliated with a teaching hospital.

The dialysis centers have structured their operations to accommodate a clientele that has low health literacy, limited English proficiency, and social factors such as lack of access to transportation that could affect adherence to dialysis treatments. The units reserve an open chair on its second and third shift to accommodate patients identified through the Epharmix intervention. The units also operate an emergency fund that covers taxi vouchers and other unexpected patient expenses.

After discussions with local dialysis centers and patients, we created a proactive short message service (SMS) and voice-messaging system, building on work done previously in telemedicine, that automatically notifies patients of upcoming dialysis sessions, provides messaging that emphasize partnership between the patient and the dialysis center, and offers a support line if any urgent issues come requiring modification or rescheduling of their session. SMS technology is commonly used to send mobile to mobile text messages in the United States and internationally. This system, hereafter referred to as “EpxDialysis,” was created under the auspices of IDEA Labs, a student-run design incubator at Washington University. In this proof of concept study, we aimed to evaluate the feasibility of utilizing the disease and population specific automated messaging system, EpxDialysis, among chronic hemodialysis patients and determining the effect of this intervention on (1) patient attendance to scheduled HD treatments, (2) subsequent patient hospitalizations and (3) cost and benefits associated with its use.

## Methods

This prospective randomized controlled study was approved by the Washington University School of Medicine Institutional Review Board. At our institution, we implemented a design-interview process to understand the reasons behind missed sessions. After consultation with nursing, administrative, and physician staff at local hemodialysis centers, we identified issues that reduced adherence to dialysis appointments. Based on this consultation, we created an SMS and voice-based messaging system to prevent HD nonadherence.

All included subjects were adult patients between the ages 18 and 75, receiving chronic hemodialysis three times per week with at least 12 weeks of prior scheduled sessions at the participating dialysis centers (Table 1). Patients were additionally required to have a history of between 2–6 missed dialysis appointments (for any cause, including hospitalization) over the prior 12-week period (out of 36 total treatments). We excluded patients who did not intend to continue hemodialysis for the subsequent 16 weeks, those without access to a mobile or landline phone, and individuals with neurocognitive disorders limiting capacity for informed consent. Eligible subjects were enrolled from two hemodialysis centers in St. Louis, Missouri during February 2015, and the methods were carried out in accordance with relevant guidelines and regulations.

**Table 1:** Patient Demographics.

Age, years, median (range)	50.0 (25–63)
Gender, n (%)	
Male	8 (42.1)
Female	11 (57.9)
Ethnicity, n (%)	
Black	16 (84.2)
White	2 (10.5)
Other	1 (5.3)
Annual income, $, median (range)	12000 (8040–76000)
Highest educational attainment, n (%)	
Some high school	4 (21.1)
High school graduate	6 (31.6)
Some college	6 (31.6)
College graduate or more	2 (10.5)
Unknown	1 (5.3)
Years on dialysis, median (range)	2.75 (0.5–10.25)

**Figure 1:**
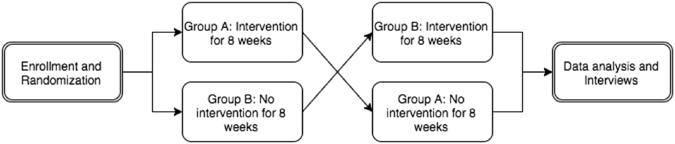
Crossover study design.

This study featured a crossover design (Fig. 1). Subjects who provided written informed consent were randomized to one of two treatment groups. Group A received the EpxDialysis automated messaging intervention for 8 weeks, followed by no intervention for 8 weeks. Group B initially received no intervention for 8 weeks, followed by the EpxDialysis messages for 8 weeks. HD standard of care was maintained for both study groups. Staff at the dialysis centers remained blinded to the group assignments of study participants.

The EpxDialysis intervention consisted of automated Short Message Service (SMS) texts or voice messages delivered to the patient’s preferred phone number three times per week. These messages provided details about the subject’s upcoming hemodialysis session, an encouraging message emphasizing the clinic’s supporting role, as well as the option for direct call routing to the dialysis center for any reason that may prevent the patient from coming to dialysis. All messages were written at an eighth-grade reading level, according to the Flesh-Kincaid Grade Level Formula (FKG score = 0.39 × (number of words/number of sentences) + 11.8 × (number of syllables/number of words) − 15.59)^18^. For example, “This is a reminder from {clinic name} about your treatment session {today/tomorrow}. We’re looking forward to seeing you. Please call us back as soon as possible at this number if you have any problems with transportation or need to reschedule.” Variations of this message rotated to reduce user fatigue, but did not differ significantly in content or length. Note that the call entered the clinic similar to any other direct patient calling a clinic, and as such the clinic was blinded as to who was in the study group.

Number of sessions missed per month was tabulated at the end of the trial and because of small available size and reported descriptively. The number of days each patient spent admitted as an inpatient was tabulated by each dialysis center from internal records. This data includes all hospitalizations for any cause and at any hospital. A conservative cost benefit analysis was performed based on the cost of disposable materials wasted as the result of each missed treatment session and expense of providing the EpxDialysis intervention to each patient per month. Costs associated with hospitalization, emergent HD and dialysis unit personnel costs were not factored into analysis.

## Results

Recruitment: 26 of 124 patients (22%) at the two dialysis centers met study inclusion criteria. Of the 94 patients excluded, 28 did not have twelve weeks of attendance history, 52 had missed fewer than two sessions in the prior twelve weeks, 11 had missed greater than six sessions, and 3 were excluded because of advanced age, neurocognitive deficits, or unavailability during the enrollment period. Table 2 shows the number of missed treatments based on attendance records over the preceding 8 weeks. Seven eligible patients did not enroll. Two patients did not enroll because they were uninterested in participating in a study, while three were uninterested in receiving messages. One patient was excluded due to neurological deficit, while one was unavailable during the enrollment period.

**Table 2:** Missed dialysis session and days hospitalized by treatment group.

Treatment group	Prior to study period (12 weeks)		During study period (8 weeks on intervention, 8 weeks on control)	
	Median number of appointments missed (n)		Median # of missed dialysis sessions	
			Intervention (n)	Control (n)
A	4 (9)		1.5 (9)	3.5 (9)
B	4 (10)		1 (10)	4 (10)
Combined	4 (19)		1 (19)	4 (19)
	**Days hospitalized (mean)** ^**A**^ **during study period**			
	**Intervention**		**Control**	
	**Average number of days hospitalized for only those that were hospitalized (n)**	**Average number of days hospitalized across the cohort (n)**	**Average number of days hospitalized for only those that were hospitalized (n)**	**Average number of days hospitalized across the cohort (n)**
A	18.0 (2)	4.4 (8)	8.0 (2)	2.0 (8)
B	8.7 (3)	2.4 (11)	7.3 (6)	4.0 (11)
Combined	12.2 (5)	3.2 (19)	7.5 (8)	3.2 (19)

HD Attendance: Through interviews with ESRD patients on hemodialysis, we determined the most common reasons for missing dialysis patients were related to symptoms of illness, lack of childcare resources and lack of transportation. Although dialysis centers may have resources including social workers and taxi vouchers, patients were often not aware of their availability. Furthermore, these dialysis patients rarely reported to the dialysis center ahead of time to notify staff of an upcoming absence, leading to waste of the medical supplies prepared for their treatment sessions. Lack of communication with the dialysis center also prevented the patients from promptly rescheduling their session to the following day or coming in for at least an abbreviated session and thereby maintaining the treatment goals or preventing unnecessary emergency dialysis sessions. In rare cases, patients unfamiliar with the symptoms of systemic fluid overload would skip sessions due to illness, without realizing that hemodialysis would relieve some of the symptoms. The inadequate two-way communication seemed to be associated with health detriments to patients and inefficient delivery of care. Prior to the use of EpxDialysis, the clinic had tried follow-up after patients missed sessions, puppet shows, and educational videos on the importance of coming to dialysis appointments. However, on interview patients reported that absences were often unplanned with extenuating circumstances as described above, and the normal flyers with the phone numbers to call for help often could not be found.

**Figure 2:**
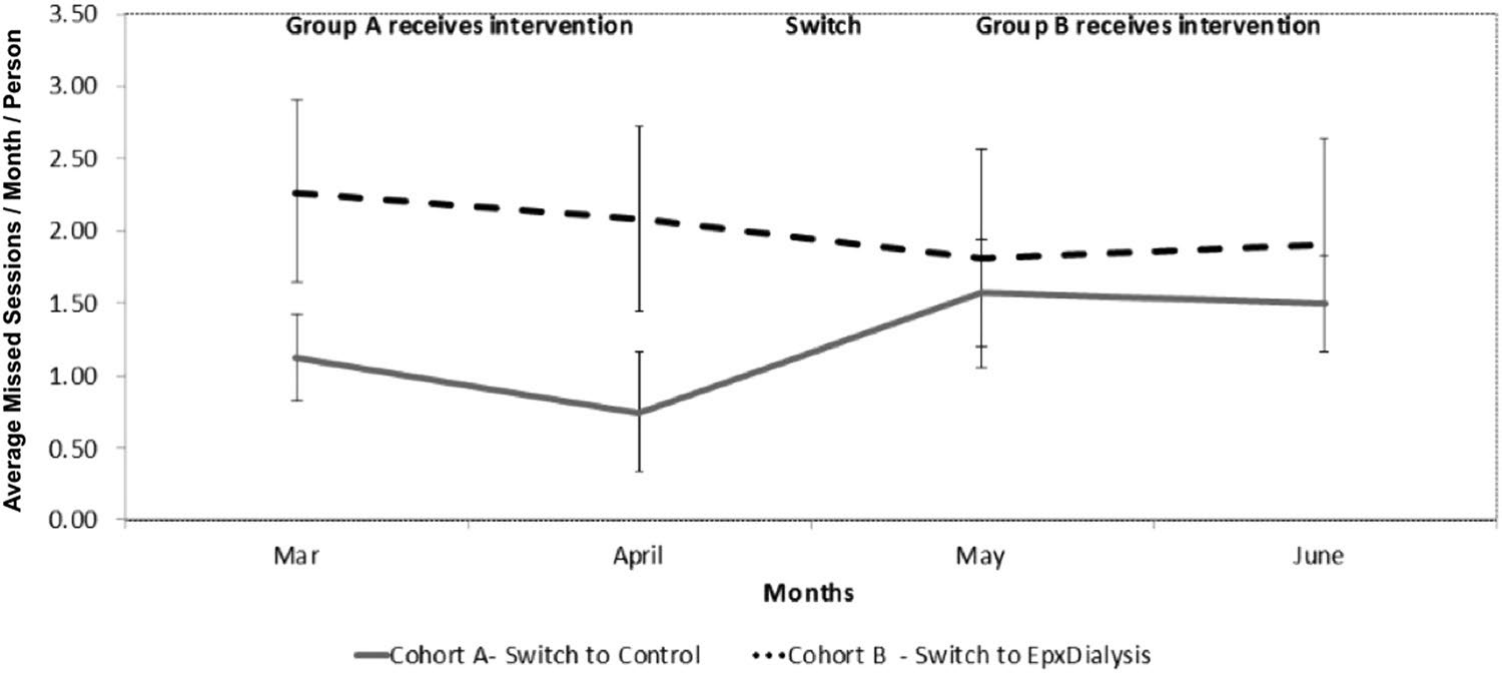
Missed appointments by treatment group and month.

With EpxDialysis, the baseline median number of missed sessions in the twelve weeks preceding the intervention was 4.5 (SE = 0.73) for group A and 4.0 (SE = 0.67) for group B. After 8 weeks of study protocol, group A (receiving the EpxDialysis) missed a median of 1.5 (SE = 0.61) sessions compared to 4.0 (SE = 1.13) for group B (control). In the eight weeks following the crossover, group A (control) missed a median of 3.5 (SE = 0.55) sessions, compared to a median of 1.0 (SE = 1.54) for group B (receiving intervention). Missed appointments by treatment group and month are outlined in Fig. 2. The pooled median number of missed sessions was 1.0 for all patients receiving the messaging intervention and 4.0 for those receiving standard of care without messaging intervention. Interviews with staff demonstrated excitement with seeing difficult patients begin to come in more frequently.

Hospitalization: Based on clinical chart review, 6 patients were hospitalized while receiving the study intervention. The median length of stay was 5.5 days. 8 patients were hospitalized during the control phase of the study, for a median duration of 8.0 days (Table 2, Fig. 1). A breakout of the days hospitalized demonstrates that the intervention did not prevent major treatment hospitalization stays (>10 days) that are secondary to underlying disease, but rather reduced the number of patients with small emergency dialysis stays <8 days (Table 3).

**Table 3:** Individual patient hospitalization data.

Patient	Days hospitalized during intervention phase	Days hospitalized during control phase
9	2	8
10	0	8
13	10	8
15	0	4
21	29	8
23	7	8
25	4	18
29	14	6
Median	5.5	8.0

Cost-Benefit analysis: Health care costs are measured by Medicare bundled services^19^, including providers, Erythropoietin (EPO), iron, vitamin D, other injectables, and certain laboratory services. A base rate of $229.63 per treatment has been assigned, to be individualized using case-mix adjusters. Assuming a conservative estimate of $50 lost in supplies per appointment missed, the total lost revenue from a patient missing dialysis is $279.63. Based on 52.5 sessions saved with the intervention, the total revenue is $13,982. At an estimated market cost for the system for $10 per patient per month, the crossover study, which provided EpxDialysis to 19 patients for 8 weeks each, incurred a total cost of $380. Therefore, the average cost to benefit ratio was 380:13,982, a 1:36 ratio.

This analysis focuses on material cost savings that directly benefit dialysis centers. If considering the effects on Medicare and Medicaid spending, the savings associated with reduced number of hospitalizations would suggest an even greater cost to benefit ratio.

## Discussion

Electronic healthcare tools involving phone communication or smart phone applications are becoming increasingly popular, however, few clinical studies have been conducted to evaluate the outcomes associated with using these tools. A recent study by Ong *et al*.^20^ highlighted the simplicity and uptake of smartphone messaging to manage complex medical conditions. Using smartphone apps to increase patients’ adherence to antihypertensive medications, the investigators noted that user adherence was high, with more than 80% of patients performing at least 80% of the recommended assessments. They also noted that mean reductions in systolic blood pressure readings between baseline and completion of their trial was statistically significant (SBP, −3.4 mm Hg; 95% CI −2.9 to −1.2). Our study intervention was the EpxDialysis system, a phone-based communication system specifically targeted towards the ESRD population on chronic hemodialysis and designed to address causes of treatment non-adherence. Consistent with previous epidemiologic studies of the ESRD population, our study population was made up of primarily non-white patients of average age 44.7 years, with lower than average income and educational attainment levels^21^. Previous work has shown this group faces psychosocial issues that limit access to care, including low health literacy and limited access to internet resources^22–24^. However, we found that phones, either landline or cell phone devices, were common among the study group. Among all patients approached for study recruitment, only 2/27 were ineligible due to lack access to a phone.

Although the factors affecting health care access of ESRD patients are complex, the problem of nonadherence with prescribed dialysis treatment sessions poses significant limits to patient health, as well as increases the costs to the health system^25^. Among the two dialysis centers evaluated, 24.2% of patients missed between 2–6 treatments in the prior 12-week period. Previous studies have shown that outcomes of ESRD are dependent on duration and regularity of hemodialysis; missing or shortening one or more dialysis sessions per month leads to increased emergency department visits and hospital admissions for emergent dialysis and acute management of ESRD-related complications, and is associated with a 30% increase in mortality^12^. According to USRDS data^1^, hemodialysis treatments cost Medicare on average $86,496 per patient per year. When sessions are missed without prior notification, the medical equipment and time of staff spent on preparing machines wasted. Due to the limited number of dialysis machines at each dialysis unit, utilization of the machines is decreased while other patients may be going without treatment. Of note, in the application of this trial, there was no change in staffing. As such, when patients called to reschedule or change their time, the absolute time dialyzed may have been reduced, but they were still able to get into the schedule with shorter wait times.

It is well known that improving adherence among hemodialysis patients is a challenging issue. Our intervention, EpxDialysis, was associated with a decrease in missed treatment sessions. Due to the cross-over design of the study, we were able to evaluate both the effects during the intervention period and after withdrawing the intervention. The pooled average number of missed appointments was 1 for patients receiving the intervention, compared to 4 for patients without intervention, representing a 75% change in attendance. A 62.5% rebound in number of appointments missed by Group A was observed when the intervention was terminated. We attribute the results observed to three components incorporated in the intervention’s design. The proactive nature of the intervention reminded patients of their upcoming session and provided guidance for clinically-concerning symptoms, such as nausea, vomiting or diarrhea. The hotline call system also prompted patients to reschedule appointments that they were unable to attend, routing any calls directly to the appropriate dialysis center. Finally, the messages, designed to include friendly greetings and communicate information at a 4th grade level, may have the additional benefit of encouraging patients to feel engaged in their care and connected to their healthcare providers.

This study demonstrated positive trends toward improved adherence with use of the intervention, however, the size of this sample represents the main limitation to this study. Due to small sample size, results could not reach statistical significance for the variables of interest. In addition, we observed high variability of results in individual patients; these stochastic effects would likely be minimized in a larger study. Another limitation is that our study was conducted from March through June, and improvements in weather allowing more accessible transport, may have contributed to higher dialysis attendance rates. However, the rebound in missed sessions observed among Group A patients during the second study period suggests that weather did not fully confound the intervention’s effect. The earlier increased effect of the system during colder months may be due to cold weather, flu season, plus the traditional difficulties with transport caused more preventable missed dialysis sessions that EpxDialysis was able to resolve. Additionally, it is possible that the staff at the dialysis centers were particularly receptive, interested, and motivated to implement systems such as ours and that staff in other settings might be less so. Though we did not measure biochemical parameters such as Kt/V and serum phosphorous that are related to adequacy of dialysis, future studies would benefit from measuring such biochemical parameters. Despite these limitations, this preliminary study indicates that EpxDialysis has the benefits of easy implementation among ESRD patients with minimal labor costs for health care facilities. Further studies featuring EpxDialysis on a larger scale are needed to investigate not only changes in patient behavior, as well as clinical outcomes, including ED admissions, hospitalizations, and death. More robust data may facilitate the improved design of EpxDialysis and offer lasting solutions for chronic hemodialysis patients.

## Conclusion

We were able to demonstrate the feasibility of implementing EpxDialysis within the chronic hemodialysis population. The trends suggest that the intervention has potential to improve adherence with medically-prescribed dialysis treatment. Though our study population was too small to undergo statistical analysis and determine statistical significance, this was an effective pilot study. These results merit future investigation of the mid and long term of effects of EpxDialysis for hemodialysis, as well as clinical outcomes associated with improved adherence. EpxDialysis is an inexpensive, low risk, non-invasive intervention that can be implemented to help high risk patients. Given the high cost-benefit ratio of 1:36, we think EpxDialysis could be a pragmatic and effective tool to improve hemodialysis appointment adherence.
